# Progress of research on the diagnosis and treatment of bone cystic echinococcosis

**DOI:** 10.3389/fmicb.2023.1273870

**Published:** 2023-10-18

**Authors:** Yibin Meng, Qian Ren, Jun Xiao, Haohao Sun, Yiping Huang, Yaqing Liu, Shan Wang, Sibo Wang

**Affiliations:** ^1^Department of Spine Surgery, Xi’an Jiaotong University Affiliated HongHui Hospital, Xi’an, China; ^2^Laboratory of Translational Medicine, School of Medicine, Shihezi University, Shihezi, China

**Keywords:** bone cystic echinococcosis, endemic disease, orthopedic surgery, neglected disease, medical advice

## Abstract

Bone cystic echinococcosis (CE) is one of the most complex and dangerous of all echinococcoses. The lack of typical imaging features and clinical manifestations makes diagnosis and treatment of this disease difficult. X-ray and computed tomography (CT) images of bone CE are similar to those of bone cysts, giant-cell bone tumors, and bone metastases, but magnetic resonance imaging (MRI) shows good diagnostic value due to excellent soft-tissue imaging features. Serological tests cannot be used as a definitive diagnostic method for bone CE due to cross-reactivity, which can lead to false-positive or false-negative results. The development of novel antigens can open new frontiers in the diagnosis of the disease. Currently, views conflict on how to diagnose and treat bone CE. Both surgical and pharmacological treatments can be used, but determining which is appropriate is difficult due to the different sites and clinical manifestations of bone CE. Radical resection is not indicated for large-bone injuries, and Pharmacotherapy becomes important. This article reviews the progress of research into the pathogenesis and clinical manifestations of, and diagnostic strategies and treatment options for, bone CE. We aimed to provide a reference for clinical diagnosis and -treatment options.

## 1. Introduction

Cystic echinococcosis, commonly called Hydatid disease is caused by the larval form of the parasitic tapeworm, Echinococcus granulosus (*E. granulosus*). Cystic echinococcosis (CE) is found on all continents except Antarctica and is classified by the World Health Organization (WHO) as one of the most neglected and geographically widespread parasitic diseases (World Health Organization, 2015). The lifecycle of *E. granulosus* involves two main hosts, one intermediate and one final. Dogs are common final hosts; the adult larvae adhere to their small intestinal mucosa, and eggs are excreted with feces. In intermediate hosts—which are humans and herbivores (cattle, sheep, goats, camels, horses, and pigs)—the eggs hatch in the body and can reach various sites via the circulatory system (Gnanasekaran et al., 2016).

Musculoskeletal involvement is rare, with an incidence of 0.5–4.0% in all CE cases (Neumayr et al., 2013a). CE can parasitize almost any bone in the body, but half of all cases occur in the spine (Loudiye et al., 2003); the incidence in other bones is lower (Schnepper and Johnson, 2004). Spinal CE was seen in all age groups, both sexes can be affected (Altinörs et al., 2000). Musculoskeletal infection of the spine often results in severe disability or even death (Inayat et al., 2019). By contrast, clinical presentations of patients with nonspinal bone CE are often nonspecific, with pain and pathological fractures being the most common (Monge-Maillo et al., 2017). Clinical history as well as laboratory, imaging, and serological tests play crucial roles in diagnosing the disease. Radical surgical resection combined with chemotherapy is the current clinical treatment of choice, but the postsurgical recurrence rate can be as high as 40% (Salman et al., 2018). Patients who experience spinal encopresis due to spinal CE often have a high recurrence rate after surgery (Caglar et al., 2019). The prognosis for patients with bone CE is poor: paraplegia, impaired mobility, postoperative disability, and even death (Gdoura et al., 2010; Arkun and Mete, 2011). Because the pathological mechanism of bone CE is unknown, the current literature consists mostly of case studies rather than systematic, comprehensive reports; therefore, consensus is lacking on the diagnosis and treatment of this disease.

## 2. Possible pathological mechanism of bone CE: hematogenous pathway and secondary infection

The route of parasitic infection in bone CE remains unclear (Cattaneo et al., 2019). In most cases, the disease is confined to the bones and rarely infects other organs (Torricelli et al., 1990). Protoscoleces (PSCs) invade the body and, via blood circulation, usually parasitize organs other than the liver. Commonly parasitized sites are the lungs; spleen; and multiple locations in brain tissue, bones, lymph nodes, and muscles (Petra et al., 2003). Therefore, both primary hematogenous and secondary infections in other organs can cause the development and progression of bone CE.

CE appears mostly in cancellous bone. Cysts lining cancellous bone can fracture bone tissue by attacking it; the disease can also spread to invade exoskeletal structures (Papanikolaou, 2008). Possible pathological mechanisms are as follows. (1) The growing cysts compress bone tissue, causing bones to atrophy (Jacquier and Piroth, 2018). (2) Cysts invade in multiple directions along less-resistant microstructures such as the bone canal; hydatid tissue erodes and replaces bone trabeculae and then destroys and breaks through the bone cortex (Neumayr et al., 2013a,b). (3) Enlarged echinococcal cysts obstruct the vessels that nourish bone, causing ischemic necrosis (Thomas et al., 1997). (4) CE cysts directly activate the proliferation of osteoclasts, causing physiological osteolysis (Song et al., 2007). (5) Cystic invasion decreases host immunity and causes soft-tissue infiltration and fistula formation, while the resulting inflammatory reaction can lead to bone destruction with neurological and joint infection (Morris et al., 2002). (6) CE lesions can spread directly to adjacent bone tissue and destroy its bony structure (Jacquier and Piroth, 2018).

The rigid structure of bone inhibits cysts from forming an exterior membrane therein (Neumayr et al., 2013a,b). Therefore, in the early stage of bone CE, cysts grow invasively along structures that offer the least resistance, such as the bone canal, and lesions appear as irregular branches (Torricelli et al., 1990). However, late-stage intrabony cysts can break through the bone cortex and involve extraosseous structures, which lack rigidity and therefore cannot restrict cystic proliferation. In addition, soft-tissue intracapsular cysts are often accompanied by plasma exudate that invades surrounding tissues. The periosteum and articular cartilage are resistant to parasitic attack; therefore, cartilage infection is rarely reported in cases of bone CE (Morris et al., 2002).

In the spine, particularly in the thoracolumbar region, due to a dense regional vascular network and rich blood supply, cysts infiltrate vertebral cancellous bone via the vertebral artery and develop along the bone marrow cavity toward the epiphyseal plate and articular cartilage in a swollen honeycomb-like or “soap bubble” shape (Arana Iniquez, 1978). Progressive sclerotic cysts compress the vertebral body, pedicle, and lamina to varying degrees, but most of the infected tissue does not attack the intervertebral disc (IVD) due to the periosteal barrier (Schnepper and Johnson, 2004).

## 3. Clinical manifestations of three types of bone CE

**Figure 1:**
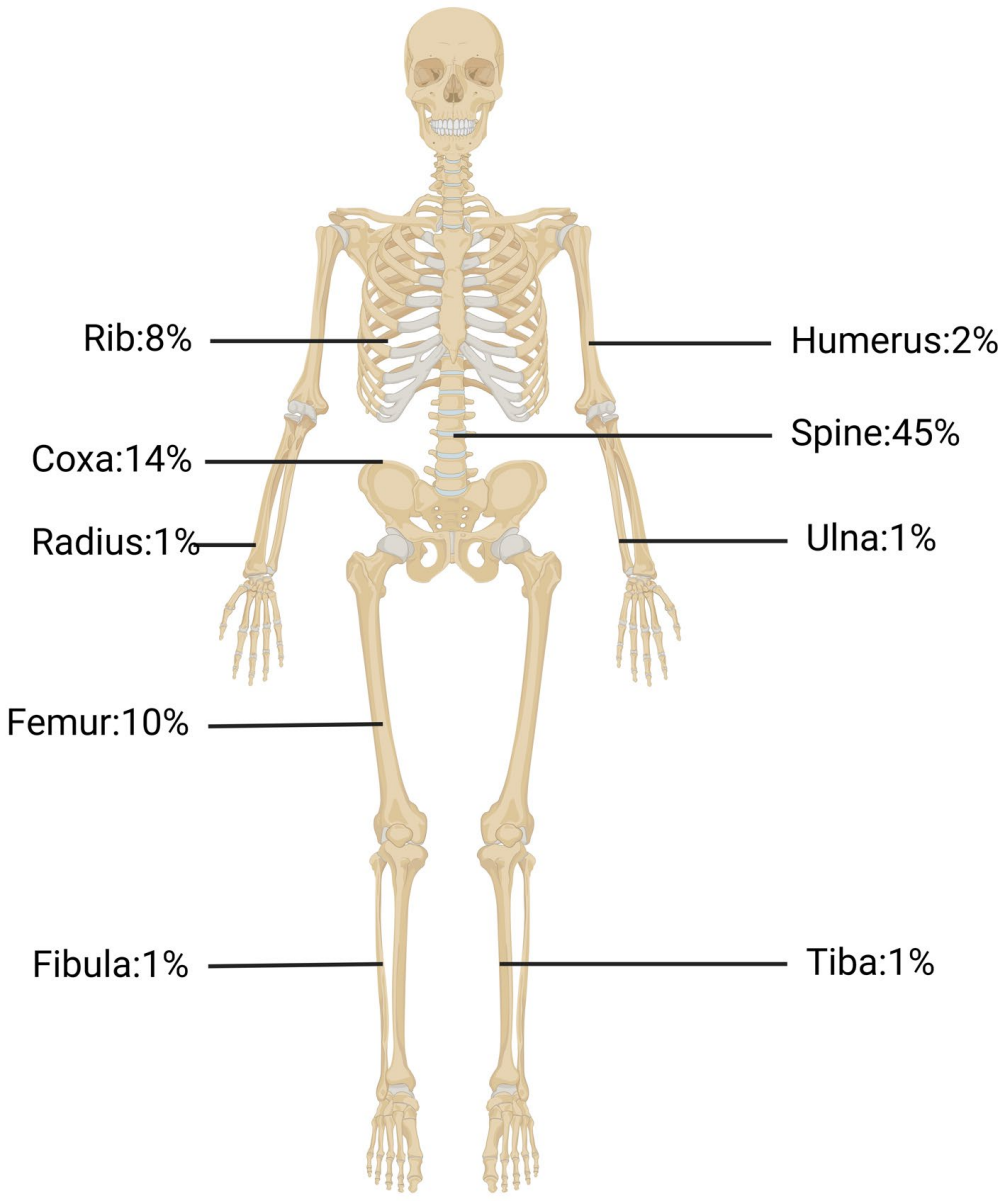
The occurrence rates of bone CE in the spine, pelvic bone, tibia bone, ribs, and other locations.

We searched the PubMed database for studies addressing recent treatment and diagnosis of bone CE and found 41 case reports thereof in the last 5 years. As shown in Table 1, the clinical presentation of bone CE is complex, with symptoms depending on the location of the infection, size of the lesion, degree of bone and surrounding-tissue invasion, and complications arising from the cyst and secondary infection (McManus et al., 2012). As shown in Figure 1, cysts can parasitize any bone in the body, but most infect only a single bone (602/721, 83%) (Steinmetz et al., 2014). The results of a European multicenter study showed that 45% of CE cases involved spinal CE and that long bones (femur, 10%; humerus; 2%) were sites of parasitization, while flat bones such as pelvic bones (14%) and ribs (8%) could also be invaded (Cattaneo et al., 2019). Echinococcosis in other parts (such as the skull, sternum, scapula and phalanx) is rare. Therefore, this article focuses on spinal CE, long bone CE and pelvic CE.

**Table 1:** The clinical manifestations of bone CE.

Classification of bone CE	Position	Manifestations
Spinal CE	Cranio-vertebral junction	Neck tilt toward the left side, neck pain, and headache along with a low-grade fever and loss of appetite (Kiran et al., 2021).
	Cervical vertebra	Sensory loss in limbs (Majmundar et al., 2019).
	Cervical and thoracic vertebrae	Walking disorders; Dull backache with paresthesia radiating down the legs (Bouattour et al., 2021); Paraparesis (Cavus et al., 2018).
	Thoracic vertebrae	Chronic back pain (Saul et al., 2020); Mid-back pain and intermittent history of fever (Das et al., 2021); Increasing thoracic pain (Depré et al., 2019); Back pain (Dighe et al., 2018); An isolated mass in the T5 vertebral body with the compression of the spinal cord (Zhang et al., 2021); Difficulty with walking and feet had no sense of cold and hot (Zhang et al., 2017). Weakness (Safari et al., 2021); Paraparesis (Akhaddar and Boucetta, 2018); Back swelling, loss of lower extremity strength, complete motor function loss, paraplegia, and immobilization (Alkan Çeviker et al., 2022); Back pain, significant weight loss, and paralysis of both lower limbs (Zhang et al., 2021).
	Thoracic vertebrae + Chest wall + Ribs	Left-sided infraclavicular chest pain and numbness in the left forearm (Hans et al., 2019); Paraparesis and back pain (Kassimi et al., 2021); Weakness and numbness of the left lower limb (Agnihotri et al., 2017).
	Lumbar vertebra	Right lower back pain and weakness in both lower limbs (Tian et al., 2020); Progressive bilateral, poorly systematized, paralyzing lumbar radiculopathy associated with urinary urgency (Staouni et al., 2020).
	Lumbar vertebra and sacral vertebra	Low-back pain (Majmundar et al., 2019); Pus discharge from the lower back; back pain; weakness of the left foot (Sharma et al., 2020); Progressive weakness of lower limbs, frequency, and urinary incontinence (Trifa and Maamri, 2021).
Long bones CE	Humeral bone	Diaphyseal humerus fracture (Patino and Ramos Vertiz, 2019).
	Radial bone	Elbow swelling (Bağcıer and Tufanoğlu, 2020).
	Ulna	Multiple swellings on the right forearm (Reddy et al., 2017).
	Femoral bone	A painful mass in the right thigh and perineal area with progressed pain and paresthesia to the right thigh and shin (Ahmady-Nezhad et al., 2022); Left groin pain and swelling in the left thigh (Salman et al., 2018); Non-union of the fracture (Gautam et al., 2018); Swelling and fracture of the left upper end of the femur; difficulty in walking and swelling in the right inguinal region (Ramteke et al., 2019); Right hip pain (Oueslati et al., 2020); Pain and swelling over her right knee region (Dathik et al., 2019); Hip pain (Masmoudi et al., 2019); Persistent thigh pain in the former fractured hip (Fröschen et al., 2019).
	Tibia bone	Pain and edema in her left upper leg (Nascimento et al., 2018).
Pelvic CE	Iliac bone	Left pelvic pain (Govindasamy et al., 2021).
	Ischiopubic branch acetabulum	Inguinal pain (Daniel et al., 2017).
	Inferior pubic ramus, ischium, and iliac bone	Left buttock pain (Dehghan Manshadi et al., 2017).
	Trochanteric region	Left hip pain (Boussaid et al., 2021).
	Pelvic bone	Progressive para-coxalgia and lower limb weakness of the left leg; pathological bone fracture (Wang et al., 2019).
	Joint space; supra-acetabular region and superior pubic ramus	Left hip pain and limp (Bhatnagar et al., 2017).
	Sacroiliac joint	Pain and paresthesia in the left gluteus (Peña Huertas et al., 2022); Left sciatica and mechanical hip pain (Akremi et al., 2022).

### 3.1. Spinal CE

The thoracic segments (46–50%) have the highest infection rate in spinal CE, and the lumbar (20–29%) and sacral (20–23%) disease also occur. The cervical spine is the least susceptible to infection of the spinal CE (Pamir et al., 2002). Eventually, spinal CE patients usually present with symptoms of spinal cord compression, with back pain in 85%, radicular symptoms in 25%, and cauda equina syndrome or even paralysis in 25–77% (Neumayr et al., 2013a). Neurological infection occurs in 20–80% of cases (Sharma et al., 2020). Patients can present with decreased sensation in one or both legs and the perineum, gradually developing signs of neurological damage such as bilateral lower-limb mobility impairments, urinary and fecal dysfunction, and weakness in urination (Sioutis et al., 2021). Ozek et al. reported that rapid-onset neurological disorders are due to inadequate blood supply caused by vascular injury; in such cases, patient recovery is often slow and incomplete (Ozek, 1994). ROBINSON RG’s case report of a female patient with severe neurological symptoms. Despite surgical treatment to remove the cyst, the patient did not have a good prognosis (Robinson, 1959). Paraplegia due to disease recurrence has been reported in as many as 45% of cases (Bhojraj and Shetty, 1999). The recurrence rate of spinal CE is 30–40%, usually due to intraoperative cyst rupture (Johnson and Hobson, 1977). Moreover, the resulting spillage of cyst contents can cause a variety of allergic reactions such as pruritus, urticaria, dyspnea, asthma, vomiting, diarrhea, abdominal cramps, bacterial infection, and even anaphylaxis. This complex clinical presentation poses great difficulties in diagnosis (Pathania et al., 2000).

### 3.2. Pelvic CE

The incidence of pelvic CE is second only to that of spinal CE. A study included 31 patients with pelvic encopresis from 1991 to 2017, with the ilium being the most common (21/31), followed by the acetabulum (7/31), pubis (6/31), sciatica (5/31), and sacrum (5/31) (Inayat et al., 2019). The pelvic bone is densely packed with cancellous bones and is rich in blood supply, providing highly favorable conditions for parasitization by *E. granulosus.* Hemipelvic infection has commonly been reported in recent years; the hip joint easily becomes infected, impairing mobility. Pelvic CE can lie latent for several years and gradually become symptomatic as the disease progresses, generally manifesting as symptoms of lumbosacral-nerve compression (Arik et al., 2015). Generally, the first clinical manifestations appear late in the disease’s progression due to the rigid skeletal structure and slow cystic growth. Severe cases are usually associated with late complications such as lumbosacral pain, swelling, fistula formation, and progressive worsening of pain in both legs (Inayat et al., 2019). Although sciatica is often reported as the first symptom of pelvic CE, it must be emphasized that the symptoms of this condition depend on the sizes and locations of cysts.

### 3.3. Long bones CE

A total of 702 patients with encopresis were included in one study, including 111 patients with long bone encopresis. The highest frequency of infection was in the femur (72/111, 65%), followed by the humerus (11/111, 10%), radius (3/111, 2.7%) and tibia (3/111, 2.7%), with ulna (1/111, 0.9%) and fibula (1/111, 0.9%) cases being rare (Steinmetz et al., 2014). As mentioned above, the femur is the most susceptible to infection. Colonization of these bones by *E. granulosus* mostly involves the epiphysis in the early stages and can initially be asymptomatic. Extensive bony lesions in later stages can lead to pathological hyperplasia of the infected limb, causing swelling and pain that becomes progressively more intense as the burden of activity increases (Song et al., 2007). Local examination of patients with femoral CE can reveal deep pressure pain in the greater trochanter, accompanied by limitation of hip motion, which can lead to late complications such as pathological fracture and fistula formation in severe cases (Kapoor et al., 2013; Inayat et al., 2019). Patients with humeral CE similarly have no obvious symptoms in the early stages. Bone erosion progresses to an advanced stage of severe bone damage, at which point patients often seek medical attention for severe pain. Although the CE of the long bones does not infect the joint surface, advanced pathological bone destruction, inflammation, and infection of the surrounding soft tissues can affect adjacent joints. Therefore, localized masses, restricted mobility, and severe pain can be clinical features of this type of CE.

## 4. Imaging examination combined with serological results to diagnose bone CE

Because the disease features of bone CE are often atypical, they often pose a diagnostic challenge to clinicians. Given that bone CE progresses very slowly, intrabony cysts can remain quiescent for long periods, even decades (Cattaneo et al., 2019). Spinal CE takes an average of at least 6 months to be diagnosed even after the onset of symptoms (Khazim et al., 2003). Imaging combined with serological testing is now the mainstay of clinical diagnosis.

### 4.1. Radiographic examination

The most commonly used imaging techniques for bone CE are X-ray, computed tomography (CT), and magnetic resonance imaging (MRI). X-ray is the first diagnostic step when patients present with pain, swelling, and other associated symptoms (Ira et al., 2001). While X-rays often do not show typical imaging features of patients with bone CE (Table 2). In addition, the periosteal reaction is usually not visible on X-ray images; if not, this indicates a pathological fracture caused by an attack on the bone cortex (Chen et al., 2020). Bone infections usually show bone destruction and cystic lesions on imaging. Cystic lesions appear as hypodensities on both CT and X-ray, but CT can show more information on these lesions, including size, extent, location, and degree of bone destruction (Tüzün and Hekimoğlu, 2001). MRI is the most relevant of all imaging modalities for the diagnosis of CE; its excellent soft-tissue resolution clearly shows the relationship between the lesion and adjacent tissues (Pamir et al., 2002).

**Table 2:** Examination of bone CE.

Classification of bone CE	Positioning	Radiological examination			Serology
		X-ray	CT	MRI	
Spinal CE	Cranio-vertebral junction (Kiran et al., 2021)	Not mentioned	An expansile lytic lesion eroding the vertebra.	Heterogeneously enhancing solid- Multiple cysts.	Not mentioned
	Cervical vertebra (Majmundar et al., 2019)	Not mentioned	Not mentioned	Cystic and enhancing lesions with significant compression.	Not mentioned
	Cervical and thoracic vertebrae (Cavus et al., 2018; Bouattour et al., 2021)	Not mentioned	Not mentioned	Multiple extradural cysts; Dura with spinal cord compression.	Negative
	Thoracic vertebrae (Zhang et al., 2017, 2021; Akhaddar and Boucetta, 2018; Dighe et al., 2018; Depré et al., 2019; Saul et al., 2020; Das et al., 2021; Safari et al., 2021; Alkan Çeviker et al., 2022)	Negative; Paraspinal opacity.	Bony nodules with lysis; The intervertebral foramen is infected and enlarged.	Spinal cord atrophy; DISC infection; Spinal cord compression.	ELISA: +; IHA: +
	Ribs (Hans et al., 2019; Kassimi et al., 2021; Agnihotri et al., 2017)	Not mentioned	Not mentioned	Par costal cyst.	ELISA: +
	Lumbar vertebra (Staouni et al., 2020; Tian et al., 2020)	Not mentioned	Vertebral body and peripheral bone hyperostotic; PET-CT: Cyst lesion on the spine.	Cyst lesion; The intervertebral foramen is infected and enlarged.	Not mentioned
	Lumbar vertebra and sacral vertebra (Majmundar et al., 2019; Sharma et al., 2020; Trifa and Maamri, 2021)	L4-L5 vertebrae were infected	Not mentioned	Multiple extradural cysts; Multiple cysts in the spinal canal.	Raised lymphocytes, eosinophil, and C-reactive proteins.
Long bones CE	Humeral bone (Patino and Ramos Vertiz, 2019)	Pathologic fracture; Multilocular cyst; Osteolytic lesions.	Not mentioned	Pathologic fracture; Multilocular cyst; Osteolytic lesions.	Not mentioned
	Radial bone (Bağcıer and Tufanoğlu, 2020)	Fracture of radius; The bony cortex thins.	Not mentioned	Hyperintense cortical destruction of bone.	Not mentioned
	Ulna (Reddy et al., 2017)	Ulnar cyst; Pathologic fracture; Soft tissue calcification.	Not mentioned	Cyst lesions with Expansive osteolytic lesions.	Not mentioned
	Femoral bone (Gautam et al., 2018; Salman et al., 2018; Dathik et al., 2019; Fröschen et al., 2019; Masmoudi et al., 2019; Ramteke et al., 2019; Oueslati et al., 2020; Ahmady-Nezhad et al., 2022)	Multiple moth-eaten-lytic areas with surrounding sclerosis in greater trochanter lesser trochanter, neck.	Soft tissue infection of bone and joint; Soft tissue calcifications.	Cystic lesions ac-cumulate in the femur; T2 hyperintense lesions.	Haemagglutinin test: positive; ELISA: IgG+
	Tibia bone (Nascimento et al., 2018)	Femoral cyst.	Not mentioned	Not mentioned	Not mentioned
Pelvic CE	Iliac bone (Govindasamy et al., 2021)	Not mentioned	Polycystic lesion of the pelvis.	Not mentioned	Not mentioned
	Ischiopubic branch acetabulum (Daniel et al., 2017)	Osteolytic lesions of the pubic acetabulum	Osteolytic lesions of the pubic acetabulum.	Cysts infect the joints and muscles.	Not mentioned
	Inferior pubic ramus, ischium, and iliac bone (Dehghan Manshadi et al., 2017)	Not mentioned	Not mentioned	Cystic lesions of the pubic ischium and ilium involving the lower limbs.	Not mentioned
	Trochanteric region (Boussaid et al., 2021)	Osteolytic lesions of the pelvis.	Not mentioned	Cystic lesions of soft tissue in the pelvis.	Not mentioned
	Pelvic bone (Wang et al., 2019)	Not mentioned	Cyst destroyed the pelvis and femoral head.	Cyst invades the periarticular muscles of the hip.	Not mentioned
	Joint space; supra-acetabular region and superior pubic ramu s (Bhatnagar et al., 2017)	Destruction of femoral head and acetabulum.	Bony destruction of the acetabular structure.	Not mentioned	ESR: up; absolute eosinophil count: up.
	Sacroiliac joint (Akremi et al., 2022; Peña Huertas et al., 2022)	Osteolytic lesions of the ischium, and fem-oral head osteonecrosis aspect.	Not mentioned	Cyst is compressing the Sciatic nerve.	Not mentioned

*The above cases information is from Table 1.

The most common manifestation of spinal CE is one or more round or oval sockets with indistinct borders that cannot be distinguished from chronic osteomyelitis on imaging (Muscolo et al., 2015). In the early stage, the lesion appears in the vertebral body and can spread to all vertebral structures. When the lesion involves the spinal canal, MRI can show the exact number and sizes of cysts, the integrity of the cyst wall, and the degree of spinal-cord compression (Herrera et al., 2005). Berk et al. reviewed the characteristics of spinal CE on MRI: (World Health Organization, 2015) sausage-like appearance with thin-walled, regular, semicircular terminals; (Gnanasekaran et al., 2016) capsular cavities without septa or fragments, occasionally spherical; (Neumayr et al., 2013a) signal intensity of capsule contents similar to that of CSF; and (Loudiye et al., 2003) capsule wall signal equal to or slightly lower than that of cystic contents on T1-weighted (T1W) images (Berk et al., 1998). In vertebral CE, the most common features are uninjured IVD and vertebral body, while the paraspinal area, subperiosteal bone, and adjacent ribs are more commonly infected (Herrera et al., 2005). Destruction of discs in the advanced stages makes spinal CE difficult to distinguish from inflammatory spinal conditions.

In pelvic CE, osteolytic cystic lesions are the single striking feature located in the ilium but can span the hip and sacroiliac joints. Vertebral osteochondral reactions are uncommon. Calcifications and cysts can be found on imaging after adjacent tissues are invaded (Rangheard et al., 2001). In long-bone CE, the primary cyst begins in the epiphysis (Dathik et al., 2019). The lesion, which can be either monocystic or polycystic, is mostly located in the metaphysis and can expand into the diaphysis and form a fan-shaped cortex; however, dilatation, sclerosis, and periosteal reaction seldom occur. Polycystic lesions are more common, presenting as large round or oval ground areas of bone destruction that collect in the epiphysis or metaphysis and greatly expand the extent of bone destruction.

The progression of bone cysts is characterized by two imaging stages: (World Health Organization, 2015) the microcystic-infiltration stage, in which the cyst creates a cluster of “grape”-like changes; and (Gnanasekaran et al., 2016) the secondary-infection stage, in which inflammatory bone disease casts a grape-like shadow of bone proliferation and destruction (Arias, 1946). In advanced stages of bone CE, the inflammatory stimulation of bone proliferation exceeds the osteolytic process, and imaging has limited specificity to distinguish the disease from bone malignancy. A study by Farrokh Saidi found that “a single cyst only,” “lamellar separation,” and “cyst degeneration” are independent predictors of good prognosis in hepatic CE (Fathi et al., 2016). However, no studies have determined whether cystic calcification can also predict prognosis in bone CE. In the author’s opinion, calcified cysts indicate a lower capacity for cystic growth, a lessened ability to invade surrounding tissues, and a tendency to limit the lesion. Nevertheless, a calcified cyst can act as an intrabony occupying lesion, compressing or even blocking the ability of intrabony trophoblastic vessels to support the bone, thereby causing bone ischemia and compressing nerve tissue in some cases.

### 4.2. Serology

Serological tests can be used to support bone CE diagnosis and as screening tools. Such tests are divided into two categories: (World Health Organization, 2015) antigen detection using encapsulated cystic fluid and PSC larvae; and (Gnanasekaran et al., 2016) detection of antibodies (aBs) in patient serum. Commonly used antigen indicators in the laboratory include anti–*E. granulosus* cyst fluid (EgCF) antigen, fine-grained echinococcal cestode antigen, epithelial glycoprotein (EGP), semi-purified CE cyst fluid antigen B (AgB), and E2 receptor alpha (Era2) (Siles-Lucas et al., 2017). In antigen-based sensitivity (Sens) and specificity (Sp) experiments, the Sen of antigen detection was 45–92% in both CE patients and healthy populations, while Spc was 70–100%. This means that the surface antigens of both populations contain similar antigenic determinant clusters, which are thought to be prone to cross-reactivity (Carmena et al., 2006). Some newer antigens, including *E. granulosus* tegumental antigen (EgTeg) and *E. granulosus* alkaline phosphatase (EgAP), have shown >90% Spc and Sen in experiments (Ortona et al., 2005). Although such results still require support from studies with large samples, they provide important reference values for the diagnosis of CE.

The sensitivity of a diagnostic test for bone CE depends on the integrity, growth viability, and locations of cysts (List et al., 2010). In the early stages, intrabony cysts are positive on serological examination due to their inability to form fibrous membranes or due to cystic rupture, infection, or abscess formation (McManus, 2014). Serological tests are mostly negative in the late stages due to cyst aging or calcification, and false-negative results cannot be avoided. The Casoni and indirect-hemagglutination tests also show good diagnostic potential for bone CE (Wang et al., 2019). Ozdemir et al. reported three cases of spinal CE; two were serologically negative but confirmed to have spinal CE via pathology (Ozdemir et al., 2004). Three problems exist with the immune response to serological diagnostic tests for CE: (1) *E. granulosus* antigens cross-react with antigens of other parasitic diseases, which can impair test specificity (2) The strength of the patient’s immune system affects serological test results, with both false-positive and -negative results occurring. (3) Test sensitivity decreases to 25–56% in extrahepatic CE (Xiao et al., 2003). Therefore, the serological examination does not provide sufficient evidence for it to be used as the main diagnostic method in bone CE and must hence be combined with other methods for comprehensive analysis.

## 5. Treatment: radical resection and drug therapy

As shown in Table 3, the treatment of bone CE is site dependent. Currently, the most appropriate treatment is radical surgery or resection of all infected bone (Arkun and Mete, 2011). However, radical surgery is difficult to perform and leaves the patient prone to recurrence, especially when the spine, pelvic bones, and ribs are infected. Therefore, surgery is sometimes combined with other treatments (e.g., radiotherapy) to prevent recurrence.

**Table 3:** Treatment of bone CE.

Classification of bone hydatid disease	Positioning	Treatment		Result
		Surgery	Drug	
Spinal CE	Cranio-vertebral junction (Kiran et al., 2021)	Decompression; resection of cyst lesion; washing with 20% hypertonic saline solution; occipital-C2- C3 vertebrae fusion	ABZ (400 mg per day for 6 weeks)	Symptoms improved after 3 months
	Cervical vertebra (Majmundar et al., 2019)	Laminectomy + resection of lesions	ABZ (pre-operation and post-operation)	Improved
	Cervical and thoracic vertebrae (Bouattour et al., 2021; Cavus et al., 2018)	Decompression; resection of cyst lesion; washing with 20% hypertonic saline solution	ABZ (15 mg/kg/d, po. For 1 year)	No recurrence after 1 year
	Thoracic vertebrae (Zhang et al., 2017, 2021; Akhaddar and Boucetta, 2018; Dighe et al., 2018; Depré et al., 2019; Saul et al., 2020; Das et al., 2021; Safari et al., 2021; Alkan Çeviker et al., 2022)	Resection of cyst lesion; ashing with betadine solution and hypertonic saline	ABZ (20 mg/kg/day for 6 months)	Improved
	Ribs (Hans et al., 2019; Kassimi et al., 2021; Agnihotri et al., 2017)	Resection of cyst lesion	ABZ	Recurred after 6 months
	Lumbar vertebra (Staouni et al., 2020; Tian et al., 2020)	Resection of cyst lesion; washing with hydrogen peroxide and 5% hypertonic saline; spinal fusion and fixation	ABZ (10-15 mg/kg/day, for at least 6 months)	Improved after 6 months
	Lumbar vertebra and sacral vertebra (Majmundar et al., 2019; Sharma et al., 2020; Trifa and Maamri, 2021)	Debridement; decompression; resection of cyst lesion; washing with hypertonic saline solution	ABZ	No recurrence for 3 years
Long bones CE	Humeral bone (Patino and Ramos Vertiz, 2019)	Oncological resection of the humerus and total replacement of the humerus	ABZ (15 mg/kg/ d) preoperation for 1 month and post-operation for 6 months	Improved
	Radial bone (Bağcıer and Tufanoğlu, 2020)	Surgery	ABZ (400 mg/d) preoperation for 1 month and post-operation for 6 months	No recurrence
	Ulna (Reddy et al., 2017)	Resection of cyst	ABZ (400 mg/d) for 6 weeks	Improved
	Femoral bone (Gautam et al., 2018; Salman et al., 2018; Dathik et al., 2019; Fröschen et al., 2019; Masmoudi et al., 2019; Ramteke et al., 2019; Oueslati et al., 2020; Ahmady-Nezhad et al., 2022)	Resection of the lesion; fixation; resection of the lesion; Reconstruction of the right hip	ABZ (400 mg, bid, proper-operation, and post-operation); praziquantel (300 mg/day post-operation)	Improved
	Tibia bone (Nascimento et al., 2018)	Resection of lesion	ABZ (10-20 mg/kg/day)	Improved
Pelvic CE	Iliac bone (Govindasamy et al., 2021)	Palliative surgery (debridement, cyst resection, 0.1% sodium hypochlorite flushing, drainage)	ABZ (pre-operation for 14 days and post-operation for 6 months)	Non-symptom
	Ischiopubic branch acetabulum (Daniel et al., 2017)	Hemipelvectomy + hip resection + hip reconstruction	ABZ (800 mg,2 doses per day, pre-operation and post-operation)	Improved
	Inferior pubic ramus, ischium, and iliac bone (Dehghan Manshadi et al., 2017)	Not mentioned	ABZ (400 mg, bid, po)	Not mentioned
	Trochanteric region (Boussaid et al., 2021)	Rejected	ABZ	Not mentioned
	Pelvic bone (Wang et al., 2019)	Cystic resection; displacement of artificial hemipelvis and hip joint	ABZ	Improved
	Joint space; supra-acetabular region and superior pubic ramus (Bhatnagar et al., 2017)	Debridement	ABZ (10 mg/kg/day; pre-operation and post-operation)	Improved; no recurrence
	Sacroiliac joint (Akremi et al., 2022; Peña Huertas et al., 2022)	Cyst resection; washing with hypertonic saline; hip replacement	ABZ	Symptom-free; No progress in disease

*The above cases information is from Table 1.

### 5.1. Surgery

Before the operation, need to determine the locations and sizes of cysts and the degree to which soft tissues surrounding the bone have been invaded. Surgical recommendations for bone CE are as follows. (1) The bones and surrounding soft tissues infected by CE must be exposed (Luan et al., 2022). (2) Integrity of the cyst wall must be ensured during resection of CE cysts (Das et al., 2021). (3) After such resection, 1–2 cm of parasite-free bone must be removed (Ozdemir et al., 2004). (4) During the operation, the surgical area should be cleaned with a short-term insecticide such as hypertonic saline to avoid recurrence caused by remaining *E. granulosus* (Khazim et al., 2003). (5) Bone grafts can be implanted for functional reconstruction after cyst resection (Thomas et al., 1997). Although many preoperative tests are available to detect osteochondroma-like lesions, bone CE is often found during surgery and confirmed by pathological examination.

Spinal CE should be given higher treatment priority than other types of bone CE. The internationally recognized classification of this disease largely guides the choice of surgical approach (Açikgöz et al., 1996). Complete resection is not possible in extensive intradural CE (Kaen et al., 2009). Intradural CE generally features multiple cysts that can attach to the lumbar-spinal roots, as well as some thin-walled cysts that can easily rupture during surgery (İşlekel et al., 1998). A limited single cyst is associated with better treatment outcomes, and surgery in such cases is considered curative if the cyst is completely removed and does not rupture (Neumayr et al., 2013a). Epidural CE lesions can vary from a single epidural worm cyst to a paravertebral encapsulated cyst to a large, dumbbell-shaped encapsulated cyst with surrounding soft-tissue invasion (Khazim et al., 2003). Patients with these two types of spinal CE are often found to have spinal cord compression. Anterior resection of the cyst is usually performed in these cases; however, if complete resection is not possible, negative-pressure aspiration and partial wall resection are desirable, and drugs with high toxicity should be avoided (Parvaresh et al., 1996). The recurrence rate of epidural CE is high (27%) because multiple cysts cannot be completely excised and the cysts are prone to rupture (Neumayr et al., 2013a).

When bone CE occurs within the vertebral body, microcystic infiltration makes complete resection difficult to achieve. However, surgical intervention can prolong patient survival (Turtas et al., 1980). Complete excision of the cyst with no destruction of the cyst wall is the standard treatment for spinal CE. However, complete cyst removal is difficult in many cases for various reasons: (World Health Organization, 2015) the cyst wall is thin; (Gnanasekaran et al., 2016) the surrounding soft tissue is attached to the cyst wall; Neumayr et al. (2013a) bone CE was not considered preoperatively; (Loudiye et al., 2003) the lesion is extensive, making surgery too invasive for the patient to tolerate; and Schnepper and Johnson (2004) surgery results in bone defects and requires the use of various techniques such as bone grafts, pedicle screw systems, titanium-cage implants, plates, or bone cement to stabilize the spine (Iplikçioğlu et al., 1991). Bone cement might be one of the best options for postoperative vertebral stabilization due to its high-temperature killing effect on PSCs, which reduces postoperative recurrence of bone CE (Yildiz et al., 2001). For large spinal-CE lesions, palliative surgical treatment plus chemotherapy might be more appropriate to limit surgical stress or damage to the patient’s neural tissues (Sudo and Minami, 2010).

Pelvic CE is the second-most widespread type of bone CE, which is difficult to treat, and the outcome and prognosis depend on whether the CE has invaded the sacroiliac or hip joints (Martínez et al., 2001). Surgical attempts to remove the lesion can fail, resulting in severe functional disability when the joint is infected. Currently, common surgical treatments for pelvic CE include simple drainage or debridement, complete resection, total hip arthroplasty, bone grafting, pubic fusion, giant prosthesis, arthroplasty, osteotomy, and hemipelvic resection (Liang et al., 2014). Hemipelvic resection is frequently used in patients with extensive pelvic CE who are infected in multiple sites. However, it is accompanied by high mortality and complications such as sepsis, pressure sores, and loss of function, meaning that patients are often resistant to this procedure. Palliative surgical treatment with long-term oral administration of effective anthelmintics such as albendazole (ABZ) is usually a good option for patients with bone CE accompanied by extensive bone destruction (Sudo and Minami, 2010). Daniel et al. reported a case of pelvic CE extending to the hip. After hip resection combined with total hip arthroplasty supplemented by perioperative medication, the patient had no signs of recurrence or sepsis at 1-year postoperative follow-up, but he required a walker as a mobility aid (Daniel et al., 2017). As can be seen, the outcome of pelvic-joint infection is very serious. Once pelvic CE infects the hip joint, it usually causes weakness in the legs and reduces joint function. Total hip replacement may be considered to restore the function of the joint.

Treatment and prognosis of long-bone CE are better than those of spinal and pelvic CE because the growth of worms is more limited in these bones than in the spine or pelvis. When the infection occurs proximally, femoral CE is more likely to infiltrate the neighboring pelvic bone. When early lesions are limited to a single segment, radical long-bone resection is the treatment of choice. If the lesion is diffusely spread, preserving the limb is not possible; amputation is the only effective treatment (Zlitni et al., 2001). Postoperative bone defects are often treated with different methods, including bone cement filling and bone grafting. Moore et al. reported a case of total femoral-replacement surgery to treat diffuse osteopathy caused by left-femoral CE, using a total femoral prosthesis (MOST Total Femoral System) to reconstruct the defect. The patient’s 1-year postoperative prognosis was good, with femoral function mostly restored (Moore et al., 2015). The use of re-aspiration (PAIR) has shown encouraging results in localized cases where surgical removal is not possible or the patient refuses surgery, relapses postoperatively, or does not respond to pharmacological treatment (Peer et al., 2023).

### 5.2. Pharmacotherapy

If PSCs infection is localized to the axial bone or if the lesion is too large, radical surgical treatment is not possible; instead, palliative surgery plus long-term medication is often the best option for improving symptoms or even curing the patient. Pharmacological treatment of CE is similar to tumor chemotherapy; ABZ can be used preoperatively to inhibit further growth of CE and even reduce cyst size (Horton, 1989), or postoperatively, either alone or in combination with other antiparasitic drugs, to prevent recurrence (Horton, 1989). However, no drugs yet exist that can effectively prevent PSCs from invading and destroying bone and muscle (Togral et al., 2016).

As shown in Table 3, drugs are an important part of perioperative bone CE management, with dosage and duration depending on the site of parasitism and degree of invasion. The action of ABZ is effective in bone CE; 10–15 mg/kg/day for at least 6 continuous months is required for better prognosis as well as a lower relapse rate. To reduce the risk of cystic-fluid rupture and its potential complications, at least 300 mg/day of praziquantel must be given in combination with ABZ. Although ABZ + praziquantel has been reported to have anti-CE activity in some cases, its efficacy remains to be further investigated in subsequent bone CE trials (Gautam et al., 2018). Postoperative chemotherapy plus surgery, a popular form of bone CE treatment in recent years, can be extended for up to 2 years in complicated cases (Agarwal et al., 1992).

One study reported a drug-loaded nanoemulsion to be similar in efficiency to ABZ in inactivating PSCs in subcutaneous tissue. The investigators concluded that the nanoemulsion had high stability, high water solubility, and greater ability to cross biomembranes, thereby proving more efficacious against lesions that were difficult to reach with ABZ (Ahmadi et al., 2020). However, validation was not performed in animal models of bone CE.

## 6. Discussion

**Figure 2:**
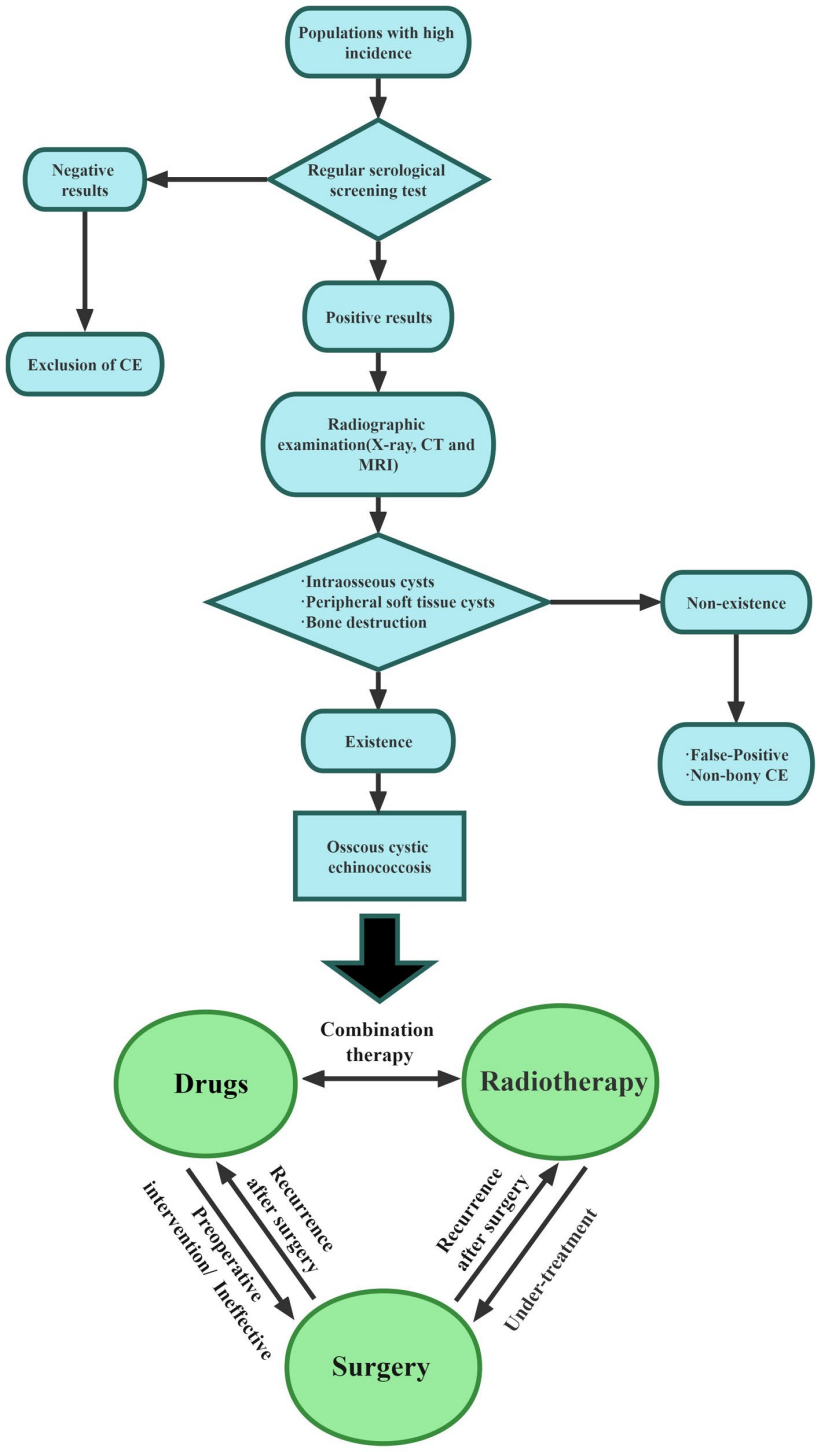
Diagnostic flow diagram of bone CE.

The research reviewed in this paper emphasizes the complexity of diagnosis and treatment of bone echinococcosis. Therefore, to understand the management of bone echinococcosis, the following aspects should be carried out. Bone CE with high rates of recurrence, disability, and paralysis, is a serious parasitic disease that imposes a severe burden on patients and families. Since bone CE mainly exists in pastoral areas, the medical level is not developed, and there is currently no clear consensus on bone CE, how to use convenient and appropriate methods for early diagnosis is undoubtedly the most important. Therefore, special medical examination centers for bone CE should be established to provide regular screening of sensitive populations and to regularly monitor the musculoskeletal conditions of vulnerable individuals. The clinical symptoms of bone CE are less pronounced in the early stages and become apparent in the later stages. Symptoms of bone CE are related to the location of the lesion and its severity. In spinal CE specifically, pain is the earliest symptom and can be accompanied by neurological manifestations. Early diagnosis and treatment are important for improving bone quality and avoiding complications, Figure 2 provides a diagnostic flow chart based on the 2015 Chinese Journal of Surgery expert consensus on the diagnosis and treatment of bone CE, hoping to provide a reference for the management of bone CE (Orthopaedics Professional Committee of Xinjiang Medical, 2015). The use of improved serological methods and new antigen development has undoubtedly improved the specificity and sensitivity of diagnosis, but there is a lack of large sample verification, which needs to be combined with imaging results. MRI is undoubtedly the most suitable imaging examination. The ‘bone window ‘and ‘soft tissue window ‘are the most sensitive for the diagnosis of bone CE. Therefore, new serological tests combined with imaging results can yield greater diagnostic value. Radical surgery combined with filler PMMA as the treatment of choice for bone CE not only repairs bone defects but can also kill PSCs. However, patients with large-bone defects often refuse radical surgery, and the risk of cystic-fluid leakage is high in such procedures due to cyst location, cyst depth, and degree of bone infiltration. Palliative surgical treatment improves patient survival while relieving the symptoms. Surgery combined with antiparasitic drugs (ABZ, praziquantel) can be used for complex manifestations of bone CE, as a chronic disease management, through systematic treatment, control and avoid complications.

## Author contributions

SiW and ShW: Conceptualization, Writing – review & editing. HS, YH and YL: Data curation, Writing – review & editing. YM and QR: Writing – original draft, Writing – review & editing.
